# Fatal H5N1 Highly Pathogenic Avian Influenza with Retrograde Neuroinvasion in a Free-Ranging Leopard Cat (*Prionailurus bengalensis*) During a Wild Bird Outbreak in South Korea

**DOI:** 10.3390/ani16020200

**Published:** 2026-01-09

**Authors:** So-Hee Gwon, Sang-Ik Park, Hyesung Jeong, Daehun Kim, Yaemoon Son, Min-a Lee, Kwanghee Lee, Young-Jae Si, Hyun-Jun Cho, Suwoong Lee, Hyeon Jeong Moon, Gun Lee, Jaewoo Choi, Chung-Do Lee, Jun-Gyu Park, Yeong-Bin Baek

**Affiliations:** 1 Wildlife Disease Research Team, National Institute of Wildlife Disease Control and Prevention, Gwangju 62407, Republic of Korea; podungvet@korea.kr (S.-H.G.); halley@korea.kr (H.J.); kdh2570@korea.kr (D.K.); sonex@korea.kr (Y.S.); mina0404@korea.kr (M.-a.L.); lkh8719@korea.kr (K.L.); mint87@korea.kr (Y.-J.S.); joon728@korea.kr (H.-J.C.); hoffman@korea.kr (S.L.); 2 Department of Veterinary Pathology, College of Veterinary Medicine and BK21 FOUR Program, Chonnam National University, Gwangju 61186, Republic of Korea; sipark@jnu.ac.kr (S.-I.P.); udlrjs77@naver.com (G.L.); cndehsla@gmail.com (C.-D.L.); 3 Department of Veterinary Pathology, College of Veterinary Medicine, Chonnam National University, Gwangju 61186, Republic of Korea; dals404@naver.com (H.J.M.); e05311cht@naver.com (J.C.); 4 Department of Veterinary Zoonotic Diseases, College of Veterinary Medicine, Chonnam National University, Gwangju 61186, Republic of Korea; kingsalt@jnu.ac.kr

**Keywords:** highly pathogenic avian influenza, H5N1 infection, *Prionailurus bengalensis*, wildlife pathology, neuropathology, retrograde neuroinvasion, meningoencephalitis, wild birds, spillover infection, wildlife disease surveillance

## Abstract

Highly pathogenic avian influenza (HPAI) H5N1 clade 2.3.4.4b viruses spread efficiently via migratory wild birds and increasingly infect mammals. The leopard cat (*Prionailurus bengalensis*) is an endangered mesopredator in South Korea that frequents wetland–forest ecotones and overlaps with wild waterbirds, placing it at risk of exposure. We describe a fatal HPAI H5N1 infection in a free-ranging leopard cat detected through national wildlife surveillance during a period of widespread H5N1 activity in wild birds along the East Asian–Australasian Flyway. The animal showed acute neurological and respiratory signs and died shortly after rescue. H5 viral RNA was detected by RT-qPCR in all examined tissues, with the highest load in the brain, and infectious virus was isolated from the brain, bronchoalveolar lavage fluid, and nasal swab. Pathology revealed acute serofibrinous pneumonia, severe nonsuppurative meningoencephalitis, necrotizing vasculitis with thrombosis, and necrotizing enteritis with secondary mesenteritis. Immunohistochemistry demonstrated abundant viral antigen in nasal and olfactory epithelium, olfactory bulb, neurons, endothelial cells, and bronchial and bronchiolar epithelium, supporting combined olfactory and hematogenous dissemination. This clinicopathological description expands the spectrum of HPAI-associated lesions in felids and underscores the value of wild carnivores as bioindicators of avian influenza spillover in a One Health context.

## Simple Summary

Highly pathogenic avian influenza (HPAI) H5N1 viruses carried by migratory wild birds have recently caused large outbreaks and unusual spillover infections in wild and domestic mammals. The leopard cat (*Prionailurus bengalensis*) is an endangered small wild felid in South Korea that inhabits wetland–forest ecotones and frequently overlaps with wild waterbirds, making it a potential sentinel for avian influenza circulation. This case report describes a free-ranging leopard cat that died from HPAI H5N1 clade 2.3.4.4b infection and was detected through the national wildlife disease surveillance program during a period of intense H5N1 activity in wild birds along the East Asian–Australasian Flyway. Necropsy and histopathology revealed severe pneumonia, diffuse meningoencephalitis, vasculitis, and gastrointestinal and mesenteric lesions. Immunohistochemistry showed abundant viral antigen in the nasal and olfactory mucosa, olfactory bulb, brain, and respiratory epithelium, supporting combined olfactory and hematogenous dissemination. This case illustrates that wild carnivores can develop rapidly fatal disease following exposure to HPAI viruses maintained in wild birds and highlights the importance of integrated surveillance in wild birds and mammals for early detection of cross-species transmission at high-risk wetland interfaces.

## 1. Introduction

Highly pathogenic avian influenza (HPAI) A viruses are negative-sense RNA viruses of the family *Orthomyxoviridae* that are naturally maintained in wild aquatic birds. Historically, low pathogenic avian influenza viruses circulated in waterfowl of the orders Anseriformes and Charadriiformes, which act as primary reservoirs and disseminate viruses along migratory flyways [1,2]. The emergence of the A/goose/Guangdong/1/96 (Gs/Gd) H5N1 lineage in 1996 and subsequent human infections in Hong Kong in 1997 marked a turning point in the ecology and public health relevance of avian influenza viruses.

Since then, Gs/Gd-lineage H5N1 viruses have diversified into multiple genetic clades and achieved global spread. Subclade 2.3.4.4b was first detected in Eurasia in late 2019–2020 and, by 2021–2022, had driven extensive epizootics in wild and domestic birds across Europe, Asia, Africa, and North America [3,4,5,6]. Many documented mammalian spillover events have been epidemiologically linked to direct or indirect exposure to infected wild birds or their carcasses, reinforcing the central role of wild birds as long-distance disseminators of HPAI viruses and the importance of integrated wildlife surveillance along migratory flyways [7,8,9].

Felids appear particularly susceptible to HPAI H5N1 infection, as demonstrated by multiple natural and experimental outbreaks in domestic cats and captive wild felids in which exposure to infected birds or raw poultry products has resulted in severe systemic and often fatal disease [10,11,12,13]. Numerous natural and experimental infections in domestic cats and captive wild felids have documented severe respiratory and neurological disease following ingestion of infected bird carcasses or contaminated poultry products [12,13,14]. Similar severe H5N1-associated disease has also been reported in other carnivores such as red foxes fed infected bird carcasses [15], supporting the broader susceptibility of carnivores to H5N1 spillover from avian reservoirs. A hallmark of HPAI H5Nx infection in carnivores is pronounced neurotropism, which is thought to reflect efficient viral replication in the upper respiratory tract and olfactory epithelium with subsequent entry into the central nervous system via the olfactory nerve and other cranial nerves and, in some strains, across a compromised blood–brain barrier. Experimental infections in ferrets have demonstrated olfactory nerve-mediated CNS invasion and severe meningoencephalitis, while field investigations of wild carnivores such as red foxes and other species have shown that viral antigen and RNA are often concentrated in the brain with associated (meningo)encephalitis and relatively mild respiratory lesions, indicating a strong neurotropic phenotype [16,17,18,19,20,21].

In South Korea, repeated incursions of clade 2.3.4.4b H5N1 viruses have resulted in extensive outbreaks in wild birds and poultry, and more recently in companion animals. Notably, outbreaks of HPAI H5N1 infection in shelter cats in Seoul during 2023 were linked to ingestion of contaminated raw duck meat and involved viruses closely related to contemporaneous avian strains circulating in the country [22,23]. In addition, HPAI H5N1 clade 2.3.4.4b infection was recently confirmed in a free-ranging leopard cat (*Prionailurus bengalensis*), representing the first documented HPAI case in a wild mammal in South Korea [24]. Together, these reports demonstrate that HPAI viruses maintained in wild birds can spillover into both domestic and wild felids in this region.

The leopard cat is a small wild felid widely distributed across South, Southeast, and East Asia and functions as a key mesopredator in many ecosystems [25]. In South Korea, the leopard cat (*P. b. euptilura*) is designated as a Class II endangered species under the Wildlife Protection and Management Act and is increasingly threatened by habitat fragmentation, road-kills, and other anthropogenic pressures [26,27]. Because leopard cats frequently use agricultural–forest and riparian mosaics where they overlap spatially with wild waterbirds and other avian prey [27], they may serve as carnivore sentinels for pathogens that are maintained and spread by wild bird reservoirs, including HPAI viruses [11].

To date, reports of HPAI H5N1 infection in free-ranging small wild felids have been scarce, and clinicopathological descriptions integrating field context and wildlife surveillance data are particularly limited for leopard cats in South Korea. Whole-genome sequencing and phylogenetic analysis of the H5N1 clade 2.3.4.4b virus isolated from the same leopard cat described here in South Korea were recently reported in a separate genomic study [24], which focused on the genetic features and evolutionary relationships of the virus in relation to contemporary wild-bird H5N1 strains. However, that genomic report provided only limited information on the clinical course, gross lesions, histopathological changes, and tissue distribution of viral antigen in the affected animal. Documenting such cases is essential to improve our understanding of how HPAI viruses maintained in wild birds’ spillover into terrestrial carnivores and to refine risk assessments for wildlife, domestic animals, and humans. In this study, we provide a detailed clinicopathological and epidemiological description of a fatal HPAI H5N1 clade 2.3.4.4b infection in a free-ranging leopard cat, and we discuss its implications for conservation and One Health-oriented avian influenza mitigation, which integrates human, animal, and ecosystem health.

## 2. Case Description

On 16 March 2025, an adult male leopard cat exhibiting marked lethargy, convulsions, and respiratory distress was found near Seryangje Reservoir in Hwasun-gun, Jeollanam-do Province, Republic of Korea. The animal was rescued and transported to the Jeonnam Wildlife Rescue Centre but was already moribund on arrival and died during isolation on 17 March 2025, before detailed clinical examination, body temperature measurement, or blood sampling could be performed. No antemortem clinical or laboratory data were therefore available for this case. The carcass was submitted the same day to the National Institute of Wildlife Disease Control and Prevention for diagnostic evaluation within the national wildlife disease surveillance program.

**Figure 1:**
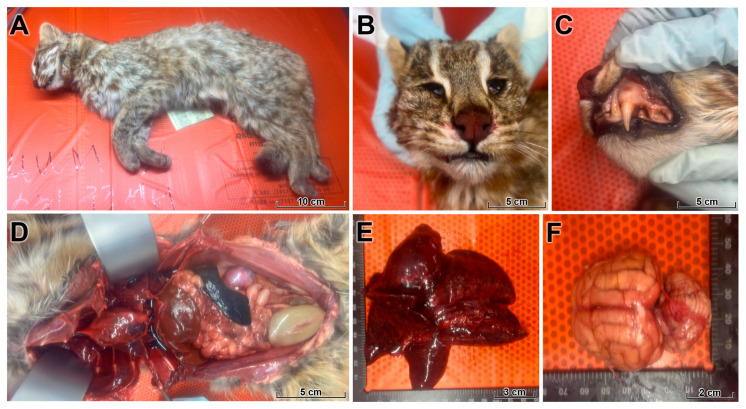
Gross findings in a wild leopard cat fatally infected with HPAI H5N1. (**A**) Whole carcass of an adult male leopard cat with moderate loss of body condition (body condition score 3/9). (**B**,**C**) Blood-tinged foamy exudate and multifocal mucosal hemorrhages in the nasal cavity and oral mucosa. (**D**) Thoracic cavity filled with a large volume of hemorrhagic effusion (hemothorax) compressing the lungs. (**E**) Lungs are heavy, fail to collapse, and show severe pulmonary edema, diffuse congestion, and multifocal pale necrotic foci affecting multiple lobes. (**F**) Dorsal view of the brain with generalized congestion, edema, and multifocal meningeal and parenchymal hemorrhages. Together, these changes indicate a fulminant systemic process with prominent vascular injury and acute respiratory failure.

At necropsy, the leopard cat weighed approximately 3.3 kg, with a body length of 70 cm and a shoulder height of 30 cm. The body condition score was estimated as 3/9, indicating moderate weight loss (Figure 1). The stomach was empty, consistent with recent inappetence or starvation. Hemorrhagic discharge was present in the nasal cavity, buccal mucosa, and gingiva.

Opening of the thoracic cavity revealed a large volume of hemorrhagic effusion (hemothorax) compressing the lungs (Figure 1). The lungs were heavy, failed to collapse, and exhibited severe pulmonary edema, diffuse congestion, and multifocal pale necrotic foci involving multiple lobes, compatible with acute influenza-associated lung injury. Examination of the cranial cavity revealed edematous, congested meninges and brain parenchyma with multifocal meningeal and parenchymal hemorrhages. Mild splenomegaly and moderate congestion of the kidneys and intestines were also observed, indicating a fulminant systemic process with prominent vascular injury.

**Figure 2:**
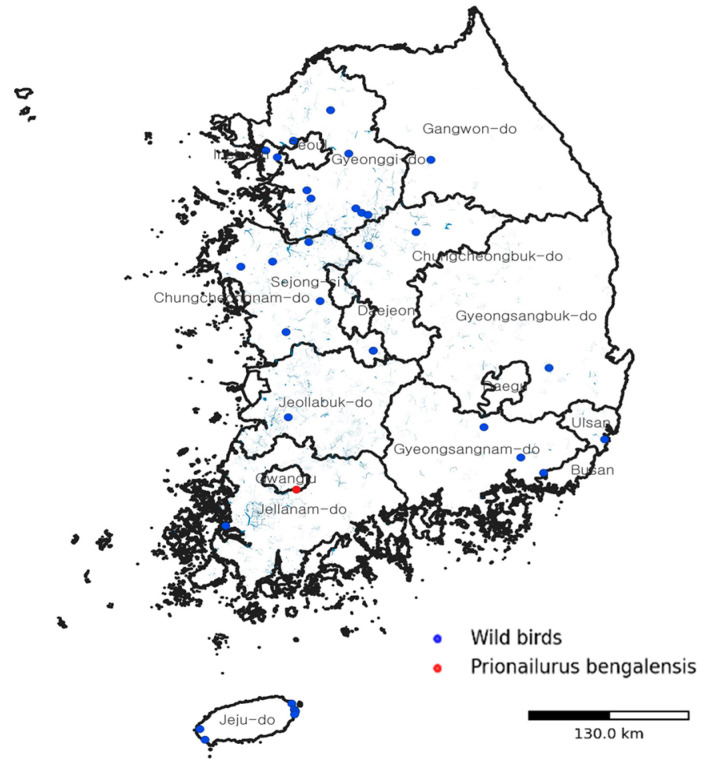
Spatial context of the HPAI H5N1 infection in a wild leopard cat in South Korea. Map showing the location where the infected leopard cat (*Prionailurus bengalensis*) was found (red circle) in relation to contemporaneous detections of HPAI H5N1 in wild birds (blue circles) during the 2024–2025 season along the East Asian–Australasian Flyway. The map illustrates how viruses maintained and disseminated by migratory wild birds intersect with the distribution of terrestrial carnivores at wetland–forest interfaces, providing ecological context for this spillover event.

Geographically, this infection occurred in an area with ongoing detections of HPAI H5N1 in migratory and resident wild birds along the East Asian–Australasian Flyway. A national overview of confirmed HPAI H5N1 cases in wild birds during the 2024–2025 season and the location of the infected leopard cat is shown in Figure 2.

All virological analyses were performed at the Wildlife Disease Control Center under the Ministry of Climate, Energy and Environment (MCEE) as part of the national avian influenza surveillance program. Tissue samples were collected from the brain, nasal turbinates, lungs, heart, liver, spleen, kidneys, pancreas, and intestines, as well as from bronchoalveolar lavage fluid (BALF) and nasal swabs. Viral RNA was extracted from tissue homogenates using an automated nucleic acid extraction platform according to the standardized surveillance protocol (see Data Availability Statement for information on access to the full protocol). Influenza A virus detection was performed by real-time reverse transcription PCR (RT-qPCR) targeting the matrix (M) gene and the hemagglutinin (HA) genes of the H5 and H7 subtypes, using validated commercial real-time RT-PCR assays. Each assay included an internal control to monitor extraction efficiency and PCR inhibition, and each run contained appropriate positive and negative controls.

H5 viral RNA was initially detected in the brain, BALF, and lungs. Subsequent RT-qPCR screening of all available samples demonstrated H5 viral RNA in every organ tested, with the lowest cycle threshold (Ct) value in the brain, indicating the highest viral load (brain Ct value, with progressively higher Ct values in BALF and lung and the highest Ct values in heart, liver, and kidney; detailed Ct values for all tissues are summarized in Table 1). Nasal swab, BALF, lung, intestine, pancreas, and spleen samples also showed diagnostically significant Ct values, whereas heart, liver, and kidney samples had higher Ct values consistent with lower viral burdens. The full distribution of Ct values across tissues is provided in Table 1 to allow direct comparison of relative viral loads.

For virus isolation, 200 µL of each sample was inoculated into the allantoic cavities of 10-day-old specific pathogen-free embryonated chicken eggs per sample following the national avian influenza surveillance standard operating procedure. Embryos were incubated at 37 °C and monitored daily for up to five days, and allantoic fluid from eggs with dead or moribund embryos was collected and tested by hemagglutination assay to confirm virus isolation. Infectious H5N1 virus was successfully isolated from the brain, nasal swab, and BALF, confirming active replication in both the central nervous system and the lower respiratory tract.

Further subtype identification was performed by RT-PCR targeting the HA and NA genes, followed by purification and Sanger sequencing of the amplicons according to the surveillance protocol. Sequence analysis identified a polybasic cleavage site (PLREKRRKRGLF) in the HA0 region, consistent with highly pathogenic H5N1 viruses of the Gs/Gd lineage.

Representative tissue samples were fixed in 10% neutral-buffered formalin, processed routinely, and stained with hematoxylin and eosin. Immunohistochemistry (IHC) for influenza A viral antigen (nucleoprotein, NP) was performed on 3 µm paraffin-embedded tissue sections using a primary antibody against influenza A NP (reference required) and a horseradish peroxidase-based polymer detection system (Dako REAL EnVision Detection System, DakoCytomation, Glostrup, Denmark). After deparaffinization and rehydration, antigen retrieval was carried out in citrate buffer (10 mM, pH 8.0), endogenous peroxidase activity was blocked with 3% hydrogen peroxide, and immunolabeling was visualized with 3,3′-diaminobenzidine followed by hematoxylin counterstaining, as previously described for IHC detection of influenza A virus antigens in mammalian tissues [28].

**Figure 3:**
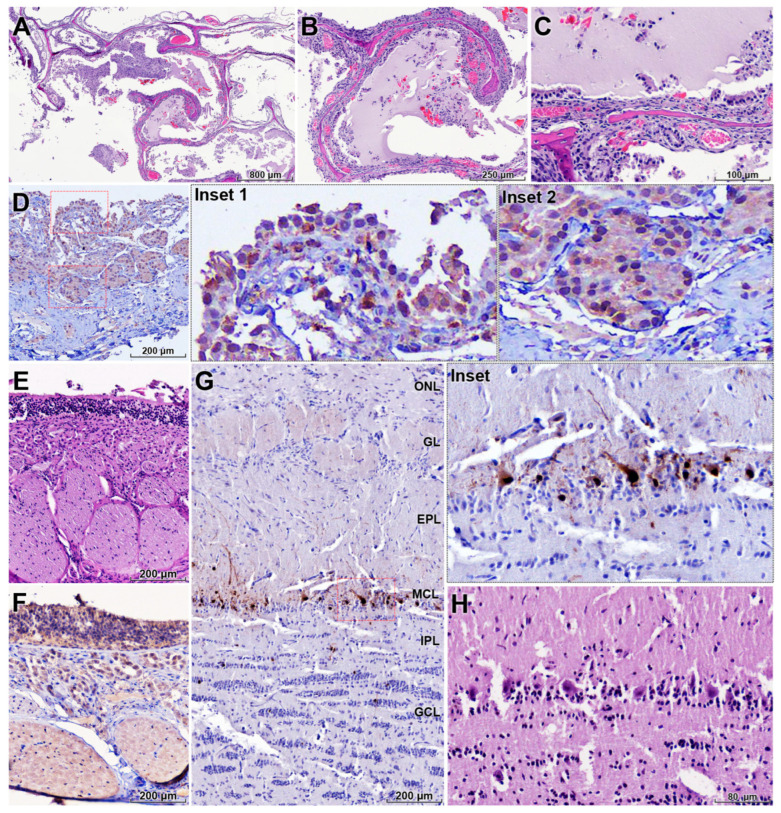
Upper respiratory and olfactory tract lesions associated with retrograde neuroinvasion of HPAI H5N1. (**A**–**C**) Nasal turbinate mucosa showing severe catarrhal to hemorrhagic rhinitis with diffuse epithelial degeneration, necrosis, and mixed lymphohistiocytic and neutrophilic inflammation (H&E). (**D**) Abundant influenza A NP antigen in necrotic respiratory epithelial cells and submucosal glands in the nasal mucosa, with marked destruction of the mucociliary apparatus (IHC). (**E**) Olfactory epithelium and Bowman’s glands with extensive epithelial necrosis and disruption of the olfactory mucosa (H&E). (**F**) Strong NP antigen labeling in the olfactory epithelium, including nerve endings of olfactory sensory neurons, indicating viral entry at the olfactory mucosa (IHC). (**G**) Olfactory bulb with prominent NP antigen localized in neurons of the mitral cell layer and adjacent neuropil (IHC). (**H**) Higher magnification of the olfactory bulb showing degenerative changes in mitral cells and spongiosis of the surrounding neuropil (H&E). These findings support retrograde spread of H5N1 along the olfactory nerve from the upper respiratory tract into the central nervous system. Red dashed boxes in (**D**) and the red frame in (**G**) indicate regions shown at higher magnification in the corresponding insets. Anatomical layers of the olfactory bulb are indicated as follows: ONL, olfactory nerve layer; GL, glomerular layer; EPL, external plexiform layer; MCL, mitral cell layer; IPL, internal plexiform layer; GCL, granule cell layer.

In the nasal cavity, the turbinate mucosa showed severe diffuse catarrhal exudate, multifocal hemorrhages, and epithelial necrosis, accompanied by lymphohistiocytic inflammation with fewer neutrophils (Figure 3A–C). IHC demonstrated abundant influenza A viral antigen in necrotic epithelial and submucosal glandular cells, with marked destruction of the mucociliary apparatus (Figure 3D).

The olfactory epithelium and Bowman’s glands exhibited extensive epithelial necrosis (Figure 3E). Strong viral antigen labeling was present in these structures and notably within the nerve endings of olfactory sensory neurons (Figure 3F). In the olfactory bulb, viral antigen was predominantly localized in neurons of the mitral cell layer (Figure 3G, inset), and affected neurons showed degenerative changes with prominent spongiosis in the adjacent neuropil (Figure 3H). These findings support retrograde viral dissemination along the olfactory nerve from the upper respiratory tract into the central nervous system.

**Figure 4:**
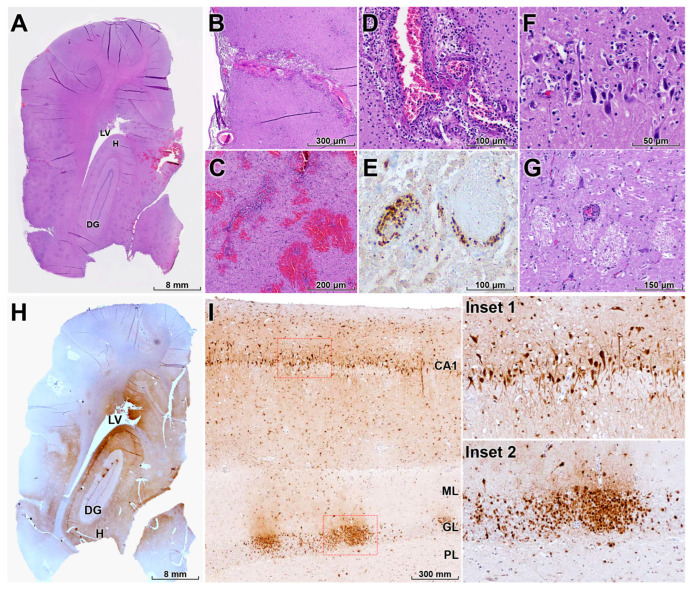
Neurotropism, vasculitis, and neuronal damage in the brain of a HPAI H5N1–infected leopard cat. (**A**) Diffuse influenza A NP antigen distribution throughout multiple brain regions, including the hippocampus (H), lateral ventricle (LV), and dentate gyrus (DG), consistent with widespread viral neuroinvasion (IHC). (**B**,**C**) Severe nonsuppurative meningoencephalitis with mononuclear inflammatory infiltrates in the leptomeninges and multifocal parenchymal hemorrhages (H&E). (**D**) Perivascular cuffs surrounding small and medium-sized vessels and segmental disruption of the vascular wall (H&E). (**E**) Necrotizing vasculitis with fibrinoid necrosis, endothelial cell damage, and intraluminal thrombosis, indicating direct vascular involvement by HPAI H5N1 (IHC). (**F**) Widespread neuronal necrosis characterized by shrunken, hypereosinophilic neurons with pyknotic nuclei, often accompanied by microglial nodules and neuronophagia (H&E). (**G**) Multifocal spongiosis with vacuolation of the neuropil between neurons and glial cells in affected brain regions (H&E). (**H**) Intense NP immunolabeling in neurons, endothelial cells, astrocytes, and microglia in the H, LV, and DG (IHC). (**I**) Red dashed boxes mark regions shown at higher magnification in the insets, which depict higher magnification of the hippocampal cornu ammonis 1 (CA1) region and DG showing heavy infection of pyramidal neurons and cells of the granule cell layer (GL); the molecular layer (ML) and polymorphic layer (PL) of the DG are also labeled as adjacent layers (IHC). Collectively, these lesions explain the severe neurological signs observed before death.

In the brain, the pattern of lesions and viral antigen distribution was most consistent with diffuse infection following retrograde neuroinvasion, likely via olfactory pathways, although concurrent or preceding hematogenous spread cannot be excluded (Figure 4A). Severe nonsuppurative meningoencephalitis was observed, extending into the leptomeninges and characterized by mononuclear inflammatory infiltrates and multifocal to locally extensive hemorrhages (Figure 4B,C). Perivascular cuffs were widespread throughout affected regions (Figure 4D). Necrotizing vasculitis was frequent, with endothelial cell necrosis and deposition of eosinophilic fibrillar material within vessel walls, consistent with fibrinoid necrosis; multiple thrombi were also present, indicating direct viral involvement of the vasculature (Figure 4D,E).

Severe diffuse neuronal necrosis was evident, with affected neurons displaying shrunken, hypereosinophilic cytoplasm and pyknotic nuclei (Figure 4F), often accompanied by neuronophagia and microglial nodules. Multifocal spongiosis, characterized by vacuolation of the neuropil between neurons and glial cells, was observed in multiple brain regions (Figure 4G). IHC revealed widespread HPAI H5N1 antigen in neurons, endothelial cells, astrocytes, and microglia, with particularly intense immunoreactivity in the hippocampus (Figure 4H). In the hippocampal CA1 region and dentate gyrus, including the granule cell layer and associated cortical layers, pyramidal neurons and cells of the dentate gyrus were heavily infected, indicating pronounced neurotropism (Figure 4I).

**Figure 5:**
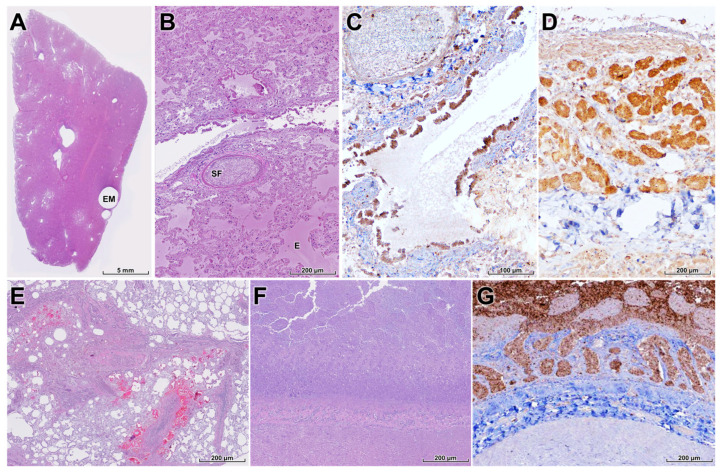
Pulmonary, mesenteric, and intestinal lesions in a wild leopard cat infected with HPAI H5N1. (**A**,**B**) Lung with multifocal emphysema (EM), severe diffuse pulmonary edema (E), serofibrinous exudation (SF), and congestion, compatible with acute respiratory distress syndrome-like injury (H&E). (**C**) Necrotizing bronchitis and bronchiolitis with extensive loss of bronchial and bronchiolar epithelium (H&E). (**D**) Strong NP antigen labeling in bronchial and bronchiolar epithelial cells and scattered alveolar cells, confirming the respiratory tract as a major site of viral replication (IHC). (**E**) Mesentery with subacute mesenteritis characterized by locally extensive fat necrosis, macrophage infiltration, and multifocal hemorrhages, interpreted as secondary to systemic vascular injury (H&E). (**F**) Small intestine showing marked villous atrophy and fusion with severe necrosis of villous enterocytes (H&E). (**G**) Diffuse crypt epithelial necrosis with NP antigen positivity in intestinal epithelium (IHC), indicating direct enteric infection that may contribute to diarrhea and poor body condition.

The lungs exhibited features of an acute respiratory distress syndrome (ARDS)-like injury, including severe diffuse pulmonary edema with serofibrinous inflammation (Figure 5A,B). There was marked epithelial necrosis in the bronchial and bronchiolar epithelium, and IHC demonstrated strong viral antigen labeling in these epithelial cells (Figure 5C,D). These lesions likely contributed substantially to respiratory failure and may have exacerbated cerebral hypoxia.

Vasculitis and venous thrombosis were frequently observed in systemic organs and were often associated with mild to moderate multifocal necrosis in the liver, pancreas, spleen, and kidneys; however, viral antigen was not detected in these organs by IHC. In contrast, severe mesenteritis was present, characterized by locally extensive fat necrosis, macrophage infiltration, and multifocal hemorrhages in the mesentery (Figure 5E). The small intestine exhibited marked villous atrophy and fusion with extensive enterocyte necrosis (Figure 5F), and the crypts showed severe diffuse necrosis attributed to direct HPAI infection, which may have contributed to progressive diarrhea and malnutrition (Figure 5G).

To investigate potential exposure sources and concurrent HPAI circulation, a field survey was conducted from 26 to 27 March 2025 around Seryang and Yonggye Reservoirs in Hwasun, within a 1–2 km radius of the location where the leopard cat was recovered. The survey focused on forest margins, agricultural fields, and riparian habitats at the wetland–forest interface.

Indirect signs (tracks, scats, feeding traces) of eight wild mammalian species were recorded: leopard cat, Eurasian otter (*Lutra lutra*), weasel (*Mustela* spp.), wild boar (*Sus scrofa*), water deer (*Hydropotes inermis*), Korean hare (*Lepus coreanus*), raccoon dog (*Nyctereutes procyonoides*), and moles (family *Talpidae*). Five presumptive leopard cat fecal samples were collected; one contained avian feather fragments, suggesting predation or scavenging of birds or bird carcasses. Avian feces were also frequently observed near mammalian activity sites, particularly around Yonggye Reservoir.

Between 17 and 25 March 2025, a total of 44 environmental and carcass-derived samples (including mammalian and avian feces and carcasses were collected from the area and tested in pooled sets for avian influenza virus by RT-qPCR; all pools were negative.

In the broader national context, between September 2024 and March 2025, 43 confirmed HPAI detections in wild birds (primarily H5N1) were reported across South Korea, mainly at key staging and wintering sites along the East Asian–Australasian Flyway (Figure 2). Concurrent carcass-based surveillance of 132 terrestrial wild mammals—including raccoon dogs, Eurasian otters, leopard cats, badgers, weasels, and martens—identified HPAI H5N1 infection only this individual leopard cat, indicating that documented spillover events in wild carnivores were relatively infrequent in this carcass-based surveillance dataset, although clustered fatal infections in leopard cats during the same outbreak period suggest that the true spillover frequency and local impact may be higher than currently recognized.

## 3. Discussion

This case report documents a fatal HPAI H5N1 clade 2.3.4.4b infection in a free-ranging leopard cat in South Korea and provides clinicopathological and epidemiological evidence linking mammalian spillover to contemporaneous wild-bird HPAI circulation. The infection occurred in an endangered mesopredator inhabiting wetland–forest ecotones and was detected within a national wildlife surveillance program that simultaneously documented widespread H5N1 activity in wild birds along the East Asian–Australasian Flyway. The same leopard cat case was previously included in a genomic report that detailed the whole-genome sequence and phylogenetic context of the H5N1 clade 2.3.4.4b virus isolated from this animal and from wild birds sampled during the same season [24]. In contrast, the present article provides the first comprehensive description of the clinical presentation, gross and histopathological lesions, tissue distribution of viral antigen, and local wildlife surveillance context for this individual, thereby complementing rather than duplicating the earlier genomic study. Together, these findings highlight how viruses maintained and disseminated by wild birds can spillover into terrestrial carnivores and underscore the importance of integrated wildlife surveillance for understanding cross-species transmission at the wildlife–domestic–human interface.

The combined virological and pathological findings indicate a fulminant systemic infection with a strong neurotropic and pneumotropic profile. H5 viral RNA was detected in all tested tissues, with the highest viral load in the brain and high loads in the respiratory tract. Infectious virus was isolated from the brain, nasal swab, and BALF, confirming active replication in both the central nervous system and lower respiratory tract.

Histologically, severe nonsuppurative meningoencephalitis, widespread neuronal necrosis, microglial activation, spongiosis, and necrotizing vasculitis with thrombosis closely resemble previously described HPAI H5Nx infections in carnivores and experimental ferret models [20,29,30]. The detection of abundant viral antigen in the olfactory mucosa, olfactory sensory neurons, and mitral cells of the olfactory bulb strongly supports retrograde neuroinvasion along the olfactory nerve as a key route of central nervous system entry. In parallel, viral antigen in endothelial cells and disseminated vasculitis and thrombosis across multiple organs suggest hematogenous spread and endotheliotropism as additional mechanisms of systemic dissemination, consistent with previous reports in cats and other carnivores [19,29].

The lungs exhibited an ARDS-like pattern characterized by severe diffuse pulmonary edema, serofibrinous exudation, and necrotizing bronchitis/bronchiolitis with robust epithelial immunolabeling. These lesions likely contributed to acute respiratory failure and may have exacerbated cerebral hypoxia, further aggravating neurological damage. Together, the respiratory and neurological signs observed clinically are consistent with the pathological changes documented at necropsy and align with descriptions of naturally occurring H5N1 infections in domestic cats and wild carnivores [12,14].

Gastrointestinal and mesenteric lesions are less frequently emphasized in HPAI H5N1 infections of carnivores [31]. In this leopard cat, we observed severe necrotizing enteritis with crypt necrosis and strong immunohistochemical labeling of viral antigen in intestinal epithelium, indicating direct enteric infection. This enteric involvement may have contributed to malabsorption, diarrhea, and the poor body condition score.

In contrast, subacute mesenteritis with fat necrosis and hemorrhage lacked detectable viral antigen, suggesting an indirect mechanism, possibly related to systemic vascular injury, ischemia, or secondary inflammatory processes. The combination of direct intestinal infection and secondary mesenteric injury broadens the recognized spectrum of HPAI-associated pathology in felids and may have implications for understanding viral shedding routes and environmental contamination in natural settings [22,31].

Although the precise route of infection in this case could not be confirmed due to the absence of stomach contents or prey remains at necropsy, several lines of evidence are consistent with exposure linked to wild birds. First, the case occurred during a period when HPAI H5N1 clade 2.3.4.4b viruses were widely detected in wild birds across South Korea, including multiple detections in waterbirds along wetlands associated with the East Asian–Australasian Flyway. Second, the infected leopard cat was found near a reservoir that serves as a habitat for migratory and resident waterbirds, and avian feces were abundant in the surrounding environment. Third, field investigation identified avian feather fragments in a leopard cat fecal sample collected within the survey area, supporting predation or scavenging of birds or bird carcasses as a plausible exposure pathway. However, the limited number of host and environmental samples collected around this case means that a wild-bird-associated spillover event cannot be demonstrated conclusively, and our inference regarding the source and direction of transmission should therefore be interpreted as probabilistic rather than definitive.

These observations are consistent with previous reports in which carnivores acquired HPAI infection through ingestion of infected birds or contaminated animal-derived products [12,14,15]. They underscore the central role of wild birds as both reservoirs and amplifiers of HPAI viruses and as key drivers of spillover at the wildlife–domestic–human interface.

From a One Health perspective, this case highlights the value of integrated surveillance systems that jointly monitor avian influenza in wild birds and wild mammals. The infection was detected as part of a coordinated national program combining fecal, live-capture, and carcass-based surveillance in wild birds with passive monitoring of mammalian carcasses. Despite extensive mammal sampling during the same period, HPAI H5N1 was only detected in this individual leopard cat, illustrating that even a single detected infection in a wild carnivore can be clinically severe and that targeted surveillance in high-risk species and habitats is important for the early detection of such events; however, given the limited number of animals examined, these data do not permit any inference about the true frequency of spillover. Integrating ecological data on wild bird migration with spatially explicit wildlife surveillance may help identify hotspots for cross-species transmission and inform risk-based interventions [32,33].

The death of an endangered leopard cat from HPAI also has important conservation implications. Populations of *P. bengalensis* in South Korea are already under pressure from anthropogenic threats, and additional disease-related mortality could further compromise local viability. Because leopard cats are mesopredators that exploit wetland–forest interfaces and interact with both wild and domestic prey species, they occupy a position where they can both acquire and potentially disseminate pathogens originating from wild birds. As such, they may serve as highly informative sentinel species for monitoring environmental circulation of HPAI viruses.

This case report has several limitations. First, detailed whole-genome sequencing and phylogenetic analysis of the H5N1 virus isolated from this leopard cat have already been reported in a separate genomic surveillance study [24], and we therefore did not repeat full-genome analyses in the present case report. Instead, our primary focus was to integrate clinical observations, gross and histopathological findings, immunohistochemical distribution of viral antigen, and the local wildlife surveillance context. Future work should combine the existing full-genome data from this and other carnivore-derived HPAI H5N1 viruses with detailed clinicopathological information to better understand their adaptation, virulence, and potential for onward transmission in mammals [34]. Second, although epidemiological and ecological evidence strongly suggests wild birds as the most plausible source of infection, direct linkage to specific avian hosts could not be demonstrated. More intensive, temporally and spatially resolved sampling of wild birds in proximity to mammalian spillover events, combined with phylogenetic analysis, will be necessary to reconstruct transmission pathways.

First, detailed genomic characterization of the H5N1 clade 2.3.4.4b virus from this leopard cat has already been reported in a separate study that performed whole-genome sequencing and phylogenetic analysis of the virus isolated from this individual [24,34]; the present work therefore did not repeat whole-genome sequencing and instead relied on targeted Sanger sequencing of the HA gene to confirm the polybasic cleavage site and pathotype. Integrative analyses that combine the in-depth pathology presented here with the previously published full-genome data, as well as future genomic data from additional carnivore cases in this region, will be important to clarify mammalian adaptation, virulence, and potential onward transmission of clade 2.3.4.4b viruses. Second, although epidemiological and ecological evidence strongly suggests wild birds as the most plausible source of infection, direct linkage to specific avian hosts could not be demonstrated. More intensive, temporally and spatially resolved sampling of wild birds in proximity to mammalian spillover events, combined with phylogenetic analysis, will be necessary to reconstruct transmission pathways.

## 4. Conclusions

This case report describes a fatal H5N1 clade 2.3.4.4b infection in a free-ranging leopard cat in South Korea, characterized by marked neurotropism, severe respiratory disease, and systemic vascular injury. The combined histopathological and virological findings support retrograde neuroinvasion via the olfactory system together with hematogenous dissemination, and reveal additional gastrointestinal and mesenteric involvement that expand the recognized spectrum of HPAI-associated pathology in felids. Occurring in an endangered mesopredator at the wetland–forest interface and in the direct context of widespread H5N1 activity in wild birds, this case exemplifies how viruses maintained and disseminated by wild birds can spillover into terrestrial carnivores and cause rapidly fatal disease.

From a One Health perspective, this leopard cat highlights the value of integrated wildlife surveillance systems that jointly monitor avian influenza in wild birds and mammals and incorporate clinical, pathological, and ecological information. Although mammalian spillover events may be relatively rare, their clinical severity and conservation impact justify targeted surveillance in high-risk species and habitats, particularly at interfaces where wild birds, domestic animals, and humans intersect. Future work should combine detailed genomic characterization of carnivore-derived H5N1 viruses with spatially explicit, multi-species surveillance to better understand adaptation, refine risk assessments, and support timely, risk-based interventions at the wildlife–domestic–human interface.

## Figures and Tables

**Table 1:** Summary of diagnostic results from selected tissues of a leopard cat infected with HPAI H5N1.

Tissue	RT-qPCR (Ct; M, H5)	Virus Isolation	Histopathological Findings
Brain	P; 9.1, 6.9	P	Severe diffuse nonsuppurative encephalitis with neuronal degeneration and necrosis, multifocal hemorrhages, and vascular thrombosis
Nasal swab	P; 25.7, 25.5	P	NA
BALF	P; 28.4, 28.4	P	NA
Lung	P; 22.6, 23.3	NA	Severe necrotizing bronchitis and bronchiolitis with diffuse edema, congestion, hemorrhages, and vascular thrombosis
Spleen	P; 30.0, 29.0	NA	No specific lesions
Kidney	P; 38.1, 37.0	NA	Mild diffuse tubular necrosis with mild vascular thrombosis
Heart	P; 31.0, 30.8	NA	Mild multifocal myocardial necrosis with mild vascular thrombosis
Liver	P; 33.4, 32.4	NA	Moderate multifocal hepatic necrosis
Intestine	P; 31.0, 29.9	NA	Severe locally extensive enterocyte necrosis
Pancreas	P; 28.0, 26.9	NA	Mild multifocal pancreatic necrosis with mild vascular thrombosis

Abbreviations: P, Positive for a test result; BALF, Bronchoalveolar lavage fluid; NA, not applied.

## Data Availability

The data presented in this study are available on reasonable request from the corresponding author. The underlying datasets and the full national avian influenza surveillance standard operating procedure cannot be made publicly available because they form part of an internal governmental surveillance program and are subject to legal and biosafety restrictions imposed by the Ministry of Climate, Energy and Environment.
